# Water Purification Efficiency and Membrane Fouling Behavior of Ceramic Membrane-Nanofiltration in Treating Water Treatment Plant Production Wastewater

**DOI:** 10.3390/membranes15120387

**Published:** 2025-12-18

**Authors:** Yawei Xie, Zewei Liu, Jiayi Yu, Zizhang Shan, Hongyuan Liu, Yan Zhang

**Affiliations:** 1 College of Civil Engineering, Zhejiang University of Technology, Hangzhou 310032, China; xyw@zjut.edu.cn (Y.X.); 221123060108@zjut.edu.cn (Z.L.); 202205130627@zjut.edu.cn (J.Y.); 221123060127@zjut.edu.cn (Z.S.); 2 College of Civil Engineering and Architecture, Zhejiang University, Hangzhou 310058, China; zhangyan@zju.edu.cn

**Keywords:** ceramic membrane-nanofiltration, production wastewater, treatment efficiency, membrane fouling analysis

## Abstract

To mitigate the risks associated with production wastewater from water treatment plants, this study evaluated the effectiveness of nanofiltration (NF) and a hybrid ceramic membrane–nanofiltration (CM–NF) process in removing natural organic matter (NOM) and Ca^2+^. A comprehensive analysis of changes in specific flux and fouling resistance of the NF membrane, combined with scanning electron microscopy (SEM) observations, provided deeper insight into membrane fouling behavior. The results show that the CM–NF process achieved average removal rates of 95.60% for DOC, 98.55% for UV_254_, 34.50% for conductivity, and 50.71% for Ca^2+^. These values represent improvements of 4.70%, 1.40%, 16.37%, and 10.36%, respectively, compared to the standalone NF process. Furthermore, CM pretreatment consistently optimized the performance of the nanofiltration system. After continuous operation, the average specific membrane flux of the CM–NF system reached 0.715, 0.67, and 0.61 under varying pollutant concentrations—increases of 10.9%, 19.6%, and 17.3% over the standalone NF system—confirming a significant improvement in permeate flux. Under continuous operation, the average degree of irreversible fouling was markedly reduced across different pollutant concentrations—decreasing from 9.2%, 17.6%, and 23.6% for the standalone NF system to 8.9%, 15.6%, and 10.9% for the CM–NF system, which clearly demonstrates the efficacy of CM pretreatment in controlling irreversible fouling. SEM observations further corroborated that CM pretreatment effectively alleviated fouling on the NF membrane surface. Additionally, higher Ca^2+^ concentrations were found to contribute to reduced membrane fouling and enhance flux performance.

## 1. Introduction

Drinking water treatment plants inevitably generate production wastewater during treatment and operational processes. Statistics indicate that the total volume of such wastewater can account for approximately 10% of the plant’s total treated water output [1]. This wastewater typically contains various refractory organic compounds and heavy metal ions. If discharged without adequate treatment, it can cause severe and long-term harm to aquatic ecosystems.

Several pretreatment approaches are commonly employed, including direct reuse [2,3,4], indirect reuse [5], advanced oxidation processes, and membrane filtration [6]. Among these [7], direct reuse may increase the concentration of disinfection byproduct precursors. Although coagulation–sedimentation—used in indirect reuse—is effective in removing certain contaminants, its efficiency in eliminating organic matter remains limited. Advanced oxidation processes can degrade pollutants but still face challenges in scalability for practical engineering applications; their performance is also influenced by factors such as temperature and pH, and they often entail high operational costs and risks of secondary pollution [8]. In contrast, membrane filtration offers distinct advantages, including straightforward operation, high treatment efficiency, and environmental sustainability.

Nanofiltration (NF) demonstrates high rejection rates for divalent ions and shows potential for treating saline wastewater. However, its efficiency is affected by several water quality parameters, such as pH, total dissolved solids (TDS), and total organic carbon (TOC) concentration [9]. For example, Song [10]. simulated various water quality conditions by adjusting concentrations of citric acid, NaOH, inorganic salts, and humic acid (HA). They observed that when the pH exceeded 6, the removal efficiency of inorganic salts improved with increasing pH. Conversely, when the TDS concentration rose to 4.5 times the baseline value, the specific permeate flux decreased by 9.1%, and the TDS removal rate dropped by 18.21%, which was attributed to intensified concentration polarization and reduced effective transmembrane pressure. Furthermore, an equivalent increase in TOC concentration resulted in an 8.78% reduction in specific permeate flux; nevertheless, the removal rates of Mg^2+^, Ca^2+^, and TDS increased significantly by 74.13%, 48.14%, and 20.50%, respectively. Similarly, Su [11]. confirmed that when DOC increased from 0.54 mg/L to 2.43 mg/L, the specific permeate flux decreased by 5.93%, whereas the removal rates of Ca^2+^, Mg^2+^, SO_4_^2−^, and CO_3_^2−^ increased by 47.23%, 58.54%, 98.75%, and 82.59%, respectively. This phenomenon may be explained by the complexation between HA and Ca^2+^, which promotes the formation of larger molecular aggregates and enhances foulant deposition on the membrane surface, thereby increasing mass transfer resistance [12,13].

During NF treatment, the system is susceptible to organic fouling, primarily caused by the presence of natural organic matter (NOM) [14,15,16]. NOM mainly consists of humic substances, polysaccharides, and proteins. To alleviate membrane fouling, pretreatment technologies are often adopted. Among these, microfiltration (MF) and ultrafiltration (UF) have gained widespread use in water treatment due to their high efficiency in removing suspended solids (SS) and colloidal particles.

Ceramic membrane (CM) technology has attracted growing interest in the water treatment field owing to its exceptional thermal and chemical stability [17]. Ceramic membranes are manufactured from inorganic materials through high-temperature sintering, which enables them to withstand fluctuations in raw water temperature and extreme pH conditions while exhibiting strong corrosion resistance. Additionally, these membranes can be effectively cleaned using strong acid or alkali solutions, possess notable anti-fouling properties, and have a long service life—typically 15–20 years. Compared to organic polymer membranes, ceramic membranes significantly reduce the frequency of maintenance and replacement in long-term operation, thereby lowering overall operational costs. In recent years, ceramic membranes have achieved considerable progress in the removal of various organic pollutants. Owing to their excellent anti-fouling performance and high pollutant rejection efficiency, they have gradually become a preferred option in the pretreatment stage of water treatment processes [18].

The NF process exhibits high removal rates for typical contaminants in production wastewater, such as Ca^2+^ and organic matter [19,20]. Meanwhile, CM demonstrates favorable chemical stability, strong anti-fouling capacity, and ease of cleaning, effectively reducing the pollutant load entering the NF unit. As a result, the combination of ceramic membrane and nanofiltration (CM–NF) shows considerable potential for application in production wastewater treatment. This study systematically compares the treatment performance of the NF process and the combined CM–NF process for production wastewater containing NOM and Ca^2+^, and further investigates the influence of NOM and Ca^2+^ concentrations on the fouling behavior of both the NF and CM–NF processes. Through an analysis of membrane flux variations and fouling resistance, complemented by SEM-based morphological characterization, the mechanisms of composite fouling induced by organics and calcium on NF membranes are delineated. These findings are anticipated to provide critical insights into the treatment efficiency of the CM–NF process under coexisting organic–calcium conditions and the extent of mitigation of NF membrane fouling achieved by CM pretreatment.

## 2. Materials and Methods

### 2.1. Experimental Methods

**Figure 1:**
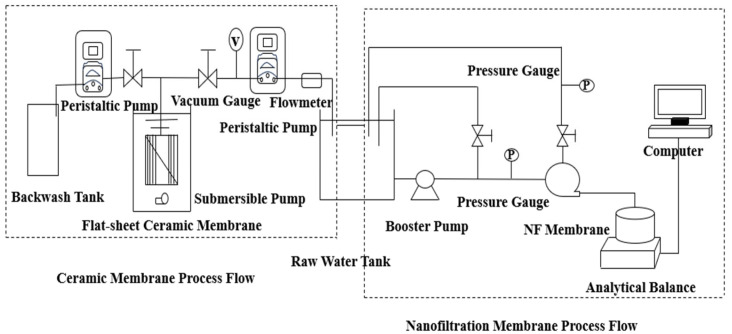
Ceramic Membrane-Nanofiltration Process.

A schematic of the experimental apparatus is shown in Figure 1. Filtration experiments using the CM were performed in a dead-end configuration under constant-flux conditions. The CM module was installed in the membrane cell, and 10 L of test water was introduced. The flux was steadily maintained at 87 L/(m^2^·h) by regulating the peristaltic pump speed, with calibration conducted every 10 min to ensure stable operation. During filtration, the transmembrane pressure (TMP) was recorded per 500 mL of permeate collected. All experiments were conducted at 25 ± 1 °C. Each experimental run concluded when the total permeate volume reached the designated value of 5 L.

The CM (Tianjian Water Affairs, Shaoxing, China) was composed of α-Al_2_O_3_, possessing a pore size of 0.1 μm and a filtration area of 275 cm^2^. The NF membrane (DuPont, Wilmington, NC, USA) was composed of a polyamide material with a nominal pore size of 1 nm and an effective area of 28.27 cm^2^. Its key characteristics included a molecular weight cut-off (MWCO) of approximately 200–400 Da, a salt rejection rate of 99.2%, a pH tolerance range of 2–11, and a maximum operating temperature of 45 °C.

NF tests were conducted using a flat-sheet test device. The NF membrane was first loaded and pre-pressurized with ultrapure water at 0.7 MPa for 2 h to stabilize its flux. Subsequently, the operating pressure was adjusted to 0.6 MPa, and the feed tank was filled with the test solution. The system was then leveled on an electronic balance to commence filtration. Throughout the experiment, the pressure was maintained constant. Permeate was collected in a beaker placed on the balance, with mass data automatically recorded by a connected computer. Membrane flux was calculated at 10-min intervals using dedicated software. Each filtration cycle lasted 8 h, representing a complete fouling-cleaning evaluation period, and the process was repeated for three consecutive cycles. At the end of each filtration cycle, a physical cleaning procedure was performed as follows: the system was operated with ultrapure water under cross-flow filtration conditions at an operating pressure of 0.6 MPa for 40 min.

### 2.2. Test Water

This study simulated production wastewater using humic acid (HA) sodium alginate (SA) and bovine serum albumin (BSA) as representative components of NOM, with CaCl_2_ (AR-grade) added to simulate water hardness [21,22]. The specific water quality parameters of the prepared synthetic wastewater containing HA (AR-grade, Shanghai Macklin, Shanghai, China), SA (AR-grade, Shanghai Macklin), BSA (98%, Shanghai Macklin), and Ca^2+^ are listed in Table 1, and the solution pH was maintained at 7.5 throughout the tests.

### 2.3. Analytical Methods

A Total Organic Carbon analyzer (RT2401A, Guangdong Puou, Shenzhen, China) was used to detect DOC content in water samples; a Fluorescence Spectrophotometer (F97, Shanghai Lengguang, Shanghai, China) was used to analyze fluorescent substances in water. A Conductivity Meter (DDS-11A, Shanghai Leici, Shanghai, China) measured the ionic conductivity of water samples; Inductively Coupled Plasma Atomic Emission Spectrometry (XU-ICP900, Shanghai Xiniu, Shanghai, China) determined the content of various inorganic ion elements in the membrane effluent; a Scanning Electron Microscope (S-4700, Hitachi Benchtop, Tokyo, Japan) was used to examine membrane surface morphology. A pH meter (model PHS-2F, Shanghai Leici, Shanghai, China) was used to measure the pH value of the water.

Changes in membrane flux can indicate the degree of membrane fouling, with units of L/m^2^·h, calculated as shown in Equation (1)

(1) $$J=\frac{V}{\mathrm{AT}}$$

where V is the filtration volume (L); A is the filtration area (m^2^); T is the filtration time (h).

Membrane fouling resistance can be divided into reversible fouling resistance and irreversible fouling resistance. Derived from Darcy’s law, the membrane flux calculation formulas are as shown in Equations (2) and (3):

(2) $$R_{1}=\frac{J_{2}-J_{1}}{J_{0}}$$

(3) $$R_{2}=\frac{J_{0}-J_{2}}{J_{0}}$$

where R_1_ is the proportion of reversible fouling layer; R_2_ is the proportion of irreversible fouling layer; J_0_ is the pure water flux before testing (L/(m^2^·h)); J_1_ is the flux at the end of filtration (L/(m^2^·h)); J_2_ is the pure water flux after backwashing (L/(m^2^·h)).

## 3. Results

### 3.1. Water Purification Efficiency

#### UV_254_, DOC, Conductivity, and Ca^2+^

**Figure 2:**
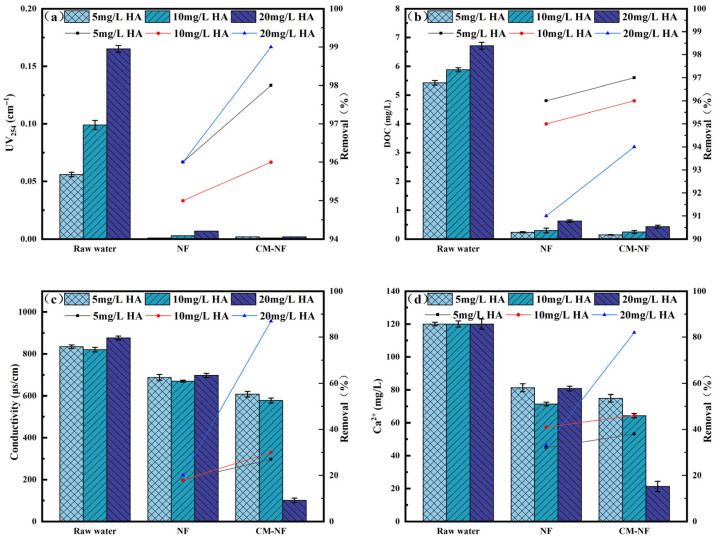
Removal effect of the process at different HA concentrations. (**a**) UV_254_. (**b**) DOC. (**c**) Conductivity. (**d**) Ca^2+^.

**Figure 3:**
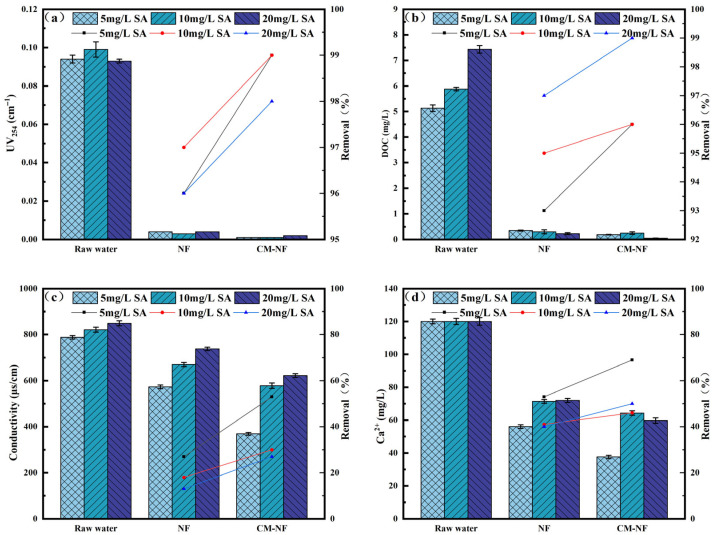
Removal effect of the process at different SA concentrations. (**a**) UV_254_. (**b**) DOC. (**c**) Conductivity. (**d**) Ca^2+^.

**Figure 4:**
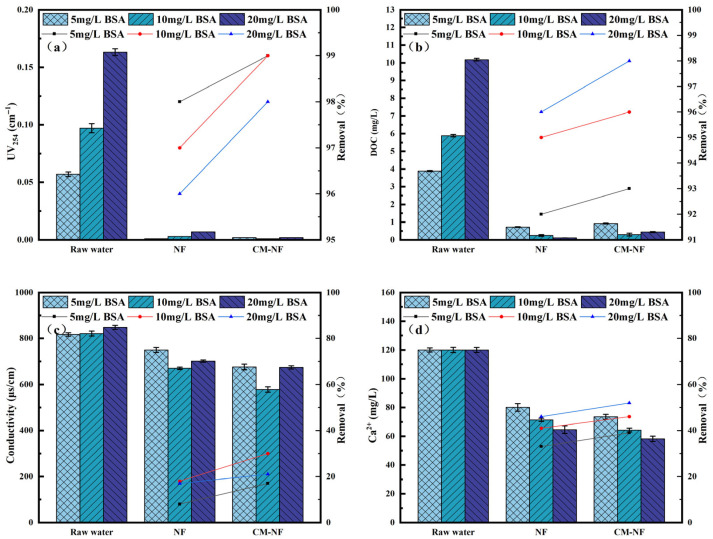
Removal effect of the process at different BSA concentrations. (**a**) UV_254_. (**b**) DOC. (**c**) Conductivity. (**d**) Ca^2+^.

The removal efficiency of UV_254_ directly reflects the retention capacity of NF and CM–NF processes for specific aromatic organic compounds [23]. The DOC removal rate serves as the primary and most straightforward indicator of a process’s overall effectiveness in eliminating dissolved organic matter. As shown in Figure 2, Figure 3 and Figure 4, in experiments involving various organic substances—including HA, BSA, and SA—the integrated CM–NF process consistently exhibited superior overall removal efficiency and operational stability compared to direct NF treatment. The average removal rates for UV_254_ and DOC were 98.55% and 95.6%, respectively, indicating that CM-NF achieved better removal performance than the ultrafiltration membranes studied by Meng [24], where the rejection rates for HA, SA, BSA, and their binary or ternary mixtures all reached above 70%. The removal of UV_254_ and DOC was mainly attributed to the multiple retention mechanisms of the NF membrane, such as steric hindrance, hydrophobic interactions, and electrostatic repulsion [25]. In addition, interactions between Ca^2+^ and different organic compounds—including complexation and gel formation—significantly influenced the removal behavior of Ca^2+^ as well as the conductivity.

Specifically, in HA systems, NF alone maintained a stable conductivity removal rate of approximately 18%, while Ca^2+^ rejection initially increased before declining, as shown in Figure 2 This trend can be attributed to the ion selectivity of the NF membrane and the Donnan effect, which favors the retention of higher-valence ions and complexes. However, organic adsorption on the membrane surface may alter its interfacial characteristics, resulting in fluctuations in ion rejection [26]. In contrast, the CM–NF process achieved significantly improved removal of Ca^2+^ and conductivity, reaching 89% and 82%, respectively, at an HA concentration of 20 mg/L.

In SA systems, CM pretreatment effectively intercepted SA–Ca^2+^ complexes, thereby mitigating the charge shielding effect on the NF membrane, as shown in Figure 3. [27]. As a result, at an SA concentration of 20 mg/L, the CM–NF process achieved removal rates of 50% for Ca^2+^ and 27% for conductivity. In comparison, direct NF exhibited lower removal efficiencies—40% for Ca^2+^ and 13% for conductivity—attributed to membrane pore blockage by gel layer formation and charge shielding.

In BSA systems, when the BSA concentration reached 20 mg/L, direct NF showed an increase in conductivity removal to 17%, while Ca^2+^ removal rose from 33% to 46%, as shown in Figure 4. This enhancement is ascribed to the nonspecific adsorption and deposition of BSA on and within the NF membrane [28], forming a denser cake layer that improved contaminant retention. Under the same conditions, CM–NF maintained stable removal rates of 97–98% for Ca^2+^ and 93% for conductivity, significantly outperforming direct NF, particularly under high BSA loading. Compared to the study by Li [21], where nanofiltration membranes based on multicomponent organic fouling comprising HA, BSA, and SA showed decreased rejection rates for NaCl (22.91–27.04%) and MgSO_4_ (64.85–83.42%) in ordinary saline and brackish water of varying concentrations, the CM–NF process demonstrated effective removal of Ca^2+^ under different concentrations of HA, SA, and BSA.

The superior performance of the CM–NF process is attributed to CM pretreatment, which modifies BSA conformation, promotes carboxyl–Ca^2+^ complexation, and partially removes pollutants prior to the NF stage [29].

**Figure 5:**
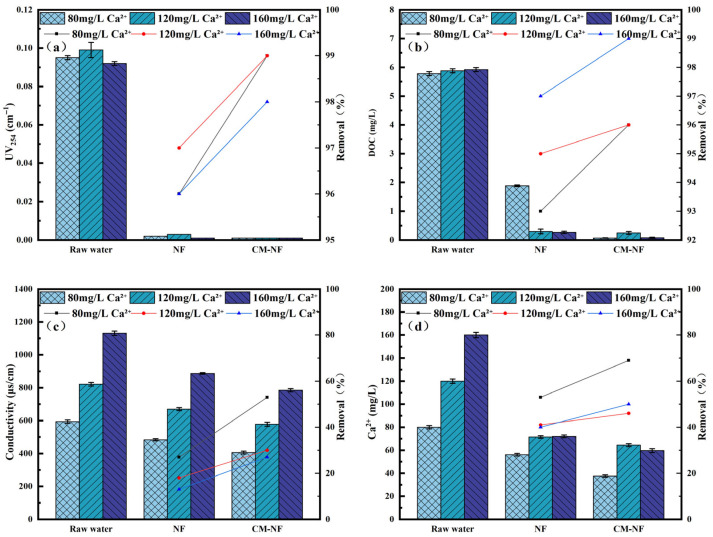
Removal effect of the process at different Ca^2+^ concentrations. (**a**) UV_254_. (**b**) DOC. (**c**) Conductivity. (**d**) Ca^2+^.

Ca^2+^ exhibited a dual role in the system, acting both as a target pollutant to be removed and as a critical factor influencing membrane fouling behavior. As shown in Figure 5, direct NF achieved only 67% DOC removal at 80 mg/L Ca^2+^, where charge neutralization dominated the interaction with organic compounds. As the concentration increased, Ca^2+^ bridging and chelation enhanced organic adsorption, raising DOC removal to 95% at 160 mg/L. Both DOC and UV_254_ removal efficiencies initially increased and then stabilized, peaking at 120 mg/L, which suggests enhanced contaminant rejection through Ca^2+^-organic complexation. However, at 160 mg/L, excessive Ca^2+^ likely induced membrane pore blockage, reducing DOC removal to 78%. Conductivity and Ca^2+^ removal remained relatively stable, possibly due to a balance between enhanced diffusion and altered electrostatic interactions caused by membrane fouling [30].

The integrated CM–NF process exhibited consistently superior and more stable performance across all evaluation indicators. This enhancement can be attributed to the graded sieving effect and pollutant configuration optimization provided by CM pretreatment, which improved feed water quality and thereby increased the overall separation efficiency of the subsequent NF stage.

### 3.2. Membrane Fouling

#### 3.2.1. Membrane Specific Flux

**Figure 6:**
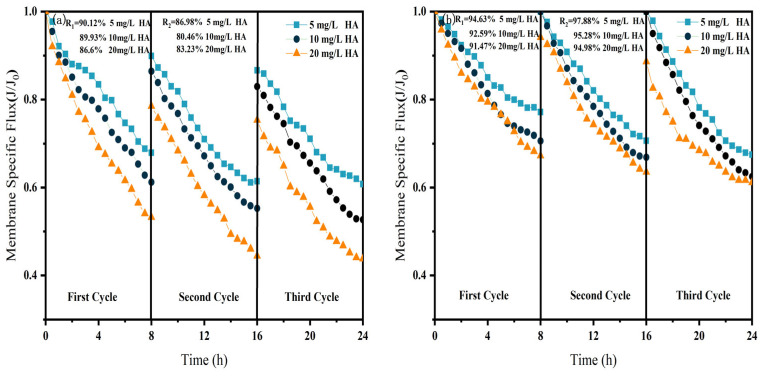
Effect of NF membrane flux at different HA concentrations (**a**) NF; (**b**) CM-NF. R_1_ and R_2_ represent the flux recovery ratios for the 1st and 2nd filtration cycles, respectively.

**Figure 7:**
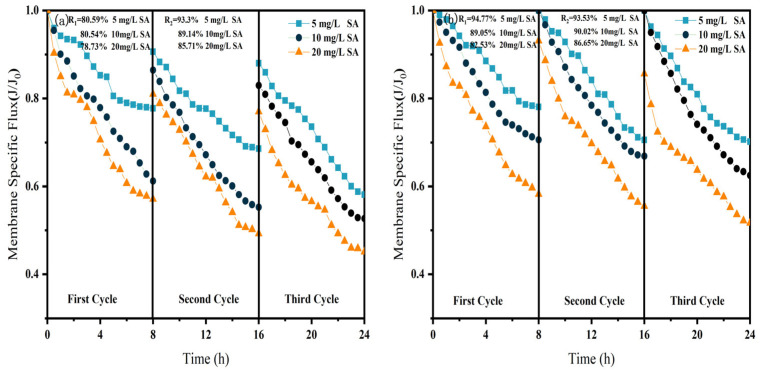
Effect of NF membrane flux at different SA concentrations. (**a**) NF; (**b**) CM-NF. R_1_ and R_2_ represent the flux recovery ratios for the 1st and 2nd filtration cycles, respectively.

**Figure 8:**
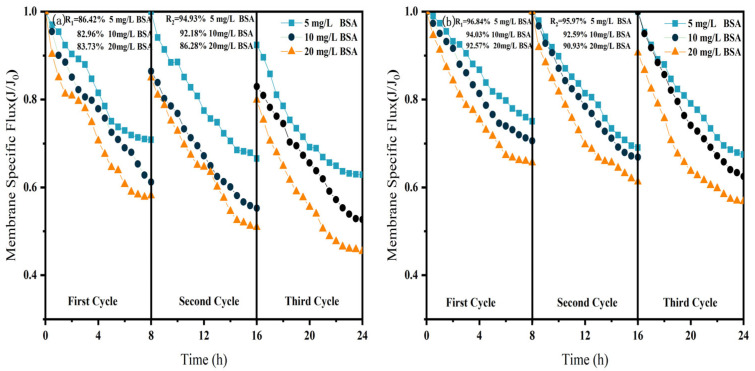
Effect of NF membrane flux at different BSA concentrations. (**a**) NF; (**b**) CM-NF. R_1_ and R_2_ represent the flux recovery ratios for the 1st and 2nd filtration cycles, respectively.

As shown in Figure 6, Figure 7 and Figure 8, in NF processes treating HA, BSA, and SA, increasing the pollutant concentration from 5 to 20 mg/L consistently exacerbated flux decline in the direct NF system. This trend manifested in three key aspects: accelerated flux decay within each filtration cycle, progressively poorer flux recovery between cycles, and a concentration-dependent reduction in the membrane-specific flux by the end of the third cycle. For instance, the specific flux declined from 0.61 to 0.44 for HA, from 0.63 to 0.45 for BSA, and from 0.58 to 0.45 for SA.

The integrated CM–NF process effectively mitigated the issues mentioned above by reducing the pollutant load entering the nanofiltration unit through ceramic membrane pretreatment. Compared to direct NF, this process significantly increased the third-cycle membrane flux by 11.5% to 38.6%, with the average membrane flux exceeding 0.6, as shown in Figure 6. In contrast, Meng [24] found that as HA concentration increased, the decline rate of the specific flux of the ultrafiltration membrane accelerated initially and then slowed after a period of ultrafiltration. After ultrafiltering 300 mL of HA solution at concentrations of 5 mg/L and 10 mg/L, the specific flux decreased by 27.3% and 37.5%, respectively, with the final membrane flux both below 0.6.

Furthermore, the fouling mechanisms and the mitigating effects of CM pretreatment differed among the organic substances. In the HA systems, fouling was mainly caused by complexation with Ca^2+^, which enhanced surface deposition; CM pretreatment reduced this effect by retaining organic matter. For SA, its inherent gelling tendency and cross-linking with Ca^2+^ resulted in the formation of a dense gel layer [31], whereas CM intercepted gel aggregates and modified the cake layer structure, increasing flux by 15.6% at 20 mg/L, as shown in Figure 7. In contrast, BSA induced irreversible fouling primarily through hydrophobic adsorption and pore blockage. In this case, CM pretreatment triggered conformational changes and aggregation of BSA [20], which alleviated pore clogging and improved flux by 26.7% at 20 mg/L, as shown in Figure 8.

In summary, the CM–NF process consistently enhanced membrane flux stability across different organic pollutant systems.

**Figure 9:**
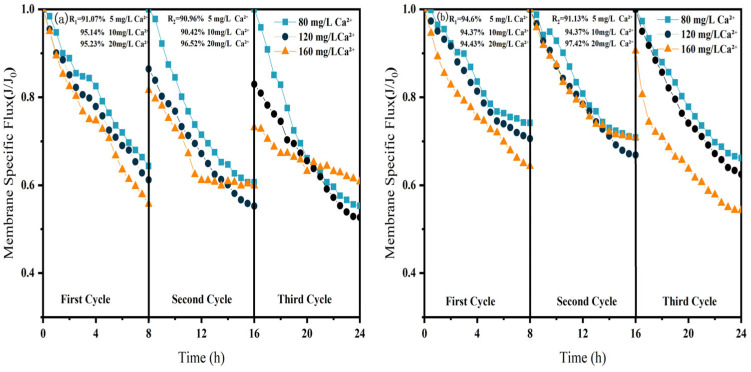
Effect of NF membrane flux at different Ca^2+^ concentrations. (**a**) NF; (**b**) CM-NF. R_1_ and R_2_ represent the flux recovery ratios for the 1st and 2nd filtration cycles, respectively.

The influence of Ca^2+^ concentration on membrane fouling exhibits characteristics distinct from those of organic pollutants, as illustrated in Figure 9. In the direct NF process, the membrane-specific flux initially decreased and then increased with rising Ca^2+^ levels: the final third-cycle flux measured 0.56, 0.52, and 0.61 at concentrations of 80, 120, and 160 mg/L, respectively. While continuous flux decline occurred throughout the cycles at lower Ca^2+^ concentrations (80 and 120 mg/L), a notable flux recovery was observed at 160 mg/L. This pattern suggests that at lower concentrations, Ca^2+^ primarily exacerbates fouling through charge neutralization, whereas at higher levels, it promotes bridging effects that facilitate organic agglomeration, thereby mitigating irreversible fouling of the NF membrane [32].

In the CM–NF integrated process, increasing Ca^2+^ concentration progressively mitigated irreversible fouling. At 120 mg/L Ca^2+^, the initial flux reached 0.88 and remained stable until the end of the second cycle, while at 160 mg/L, the flux exhibited a nonlinear variation pattern across the three cycles. These results indicate that the bridging effect induced by higher Ca^2+^ concentrations acts synergistically with the pretreatment capability of the ceramic membrane, collectively reducing fouling in the NF stage.

#### 3.2.2. Membrane Fouling Resistance

**Figure 10:**
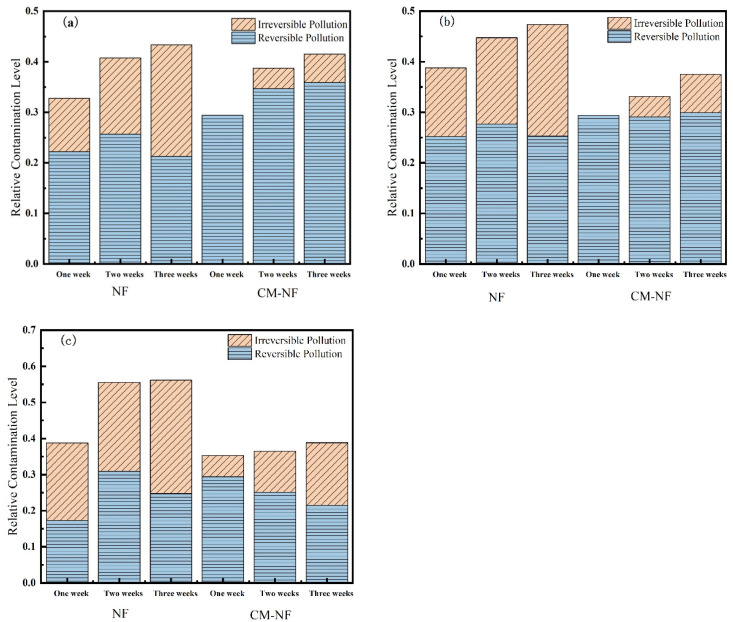
Effect on membrane resistance at different HA concentrations. (**a**) 5 mg/L; (**b**) 10 mg/L; (**c**) 20 mg/L.

**Figure 11:**
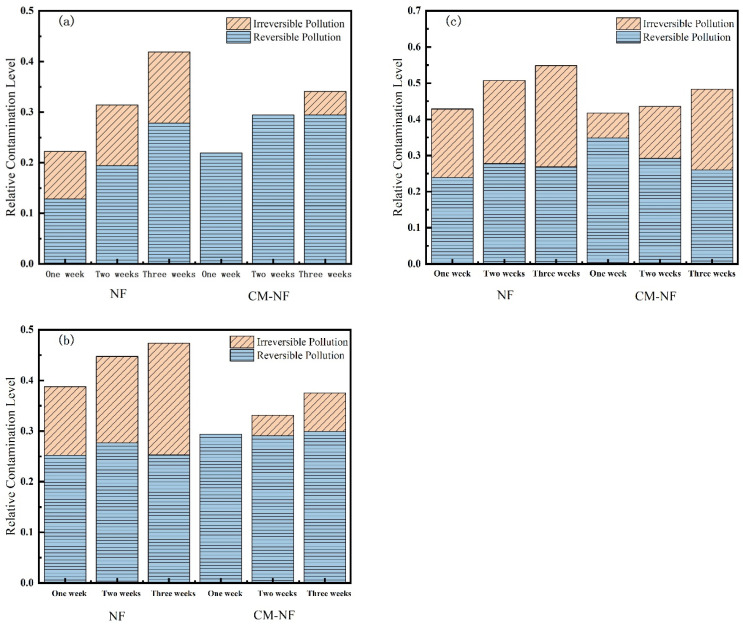
Effect on membrane resistance at different SA concentrations. (**a**) 5 mg/L; (**b**) 10 mg/L; (**c**) 20 mg/L.

**Figure 12:**
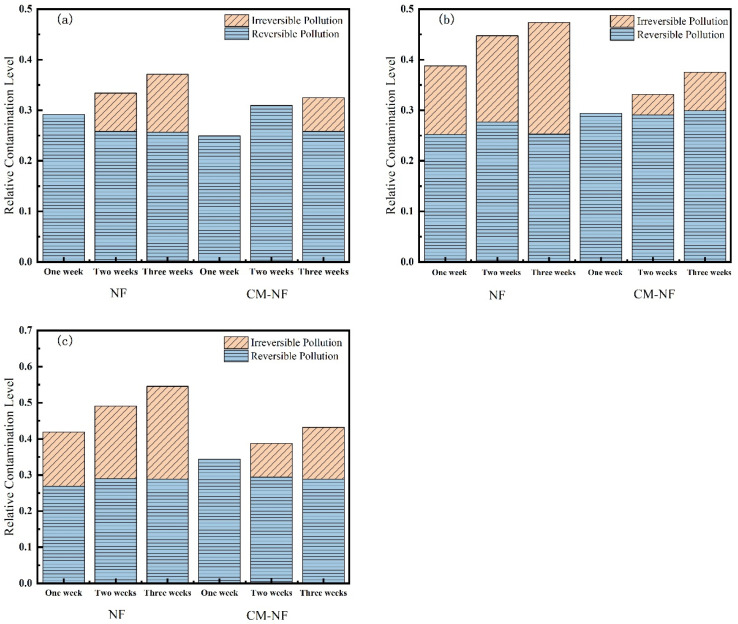
Effect on membrane resistance at different BSA concentrations. (**a**) 5 mg/L; (**b**) 10 mg/L; (**c**) 20 mg/L.

Membrane resistance is a physical quantity that quantitatively describes the degree of obstruction encountered during membrane filtration. As shown in Figure 10, Figure 11 and Figure 12, the concentration of HA, BSA, and SA significantly influenced the fouling behavior of NF membranes. In direct NF operation, elevated pollutant concentrations consistently led to increased total fouling resistance and a progressively higher proportion of irreversible fouling over multiple operational cycles. Specifically, when the HA concentration increased from 5 to 20 mg/L, the irreversible fouling ratio in the third cycle rose from 18.19% to 31.47%. Similarly, at 20 mg/L BSA, irreversible fouling reached 25.69% in the third cycle, while SA exhibited a comparable trend, with total fouling resistance reaching 47.65% at the same concentration.

The integrated CM–NF process demonstrated effective fouling mitigation across all pollutant systems. For HA at 20 mg/L, CM pretreatment reduced the irreversible fouling ratio to 17.36% in the third cycle, while making reversible fouling the dominant form (increasing from 21.48% to 29.41%). Compared to the study by Li et al. [21] on organic fouling of nanofiltration membranes with different components (HA, BSA, SA), in which reversible fouling accounted for approximately 56.0–85.1% of total fouling while the remaining irreversible fouling still occupied a considerable proportion (about 14.9–44.0%), the integrated CM–NF process can effectively alleviate membrane fouling. At the same concentration, the irreversible fouling ratio for BSA was lowered to 14.32%, representing a reduction of more than 10% compared to direct NF. In the case of SA, the CM–NF process maintained the total fouling resistance below 50% by promoting the aggregation of organic matter into more readily reversible deposits.

Notably, the fouling mechanisms varied among the different pollutants. HA primarily induced irreversible fouling through π–π interactions and chemical adsorption [33] between its aromatic structures and the membrane surface. BSA caused fouling mainly via nonspecific adsorption and pore blockage, while SA exacerbated fouling through gel formation and pore accumulation. In all cases, CM pretreatment effectively alleviated fouling through physical screening, thereby mitigating the specific fouling mechanisms associated with each type of organic pollutant.

**Figure 13:**
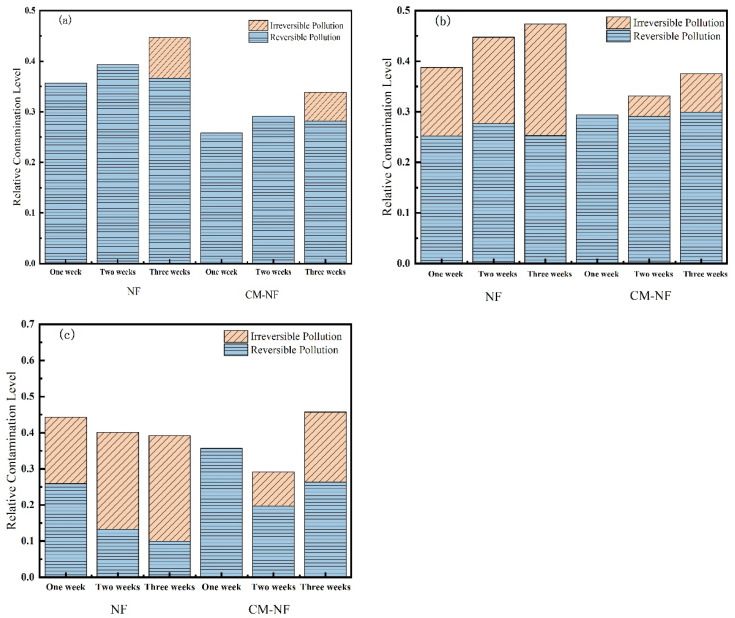
Effect on membrane resistance at different Ca^2+^ concentrations. (**a**) 80 mg/L; (**b**) 120 mg/L; (**c**) 160 mg/L.

As shown in Figure 13, Ca^2+^ concentration significantly influenced both the total resistance and fouling reversibility of the NF membrane. In the direct NF process, increasing the Ca^2+^ concentration led to an initial rise followed by a decline in total fouling resistance, while the proportion of reversible fouling exhibited a gradual decrease. Notably, at 160 mg/L Ca^2+^, fouling became predominantly irreversible. This behavior can be explained by the accumulation of Ca^2+^ on the membrane surface and within pore structures, as well as its role in bridging organic matter. These mechanisms collectively strengthen the adhesion of the cake layer to the membrane surface, thereby diminishing the effectiveness of hydraulic cleaning. Correspondingly, the total fouling resistances measured in the third cycle were 44.71%, 47.35%, and 39.18% at increasing Ca^2+^ levels, also reflecting a trend of initial increase followed by subsequent decline.

In the CM–NF process, the trend of total resistance—first increasing and then decreasing with rising Ca^2+^ concentration—was more pronounced. Notably, at 160 mg/L Ca^2+^, the irreversible fouling ratios over the three cycles were only 0%, 9.44%, and 19.38%, significantly lower than those in direct NF (18.42%, 26.88%, and 29.19%). Although irreversible fouling increased slightly over successive filtration cycles, the total resistance continued to decrease, and fouling remained largely reversible. This pattern can be attributed to two synergistic mechanisms: first, higher Ca^2+^ concentrations induce a coagulation effect that promotes the aggregation of organic matter in the feedwater; second, these larger aggregates are effectively intercepted by CM pretreatment, reducing the pollutant load entering the NF unit. Concurrently, the smaller aggregates present in the CM effluent tend to form a loose cake layer structure that is easily removed by hydraulic flushing.

### 3.3. SEM Analysis of Nanofiltration Membrane Surface

**Figure 14:**
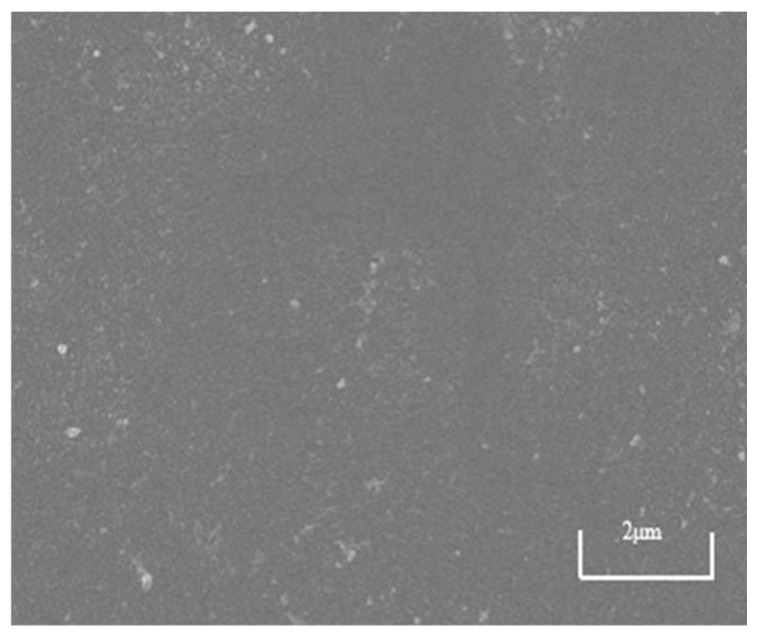
SEM image of NF270 virgin surface.

SEM images serve as the most direct and powerful substantiation for analyses of membrane fouling resistance and flux variation, achieving mutual validation between macroscopic performance and microscopic morphology. As shown in Figure 14, the surface of the NF270 membrane is smooth and flat, without visible cracks or impurities, and the membrane pores are small, requiring high magnification to observe clear pore structures. This characteristic gives it high size exclusion capability, effectively retaining larger organic molecules and exhibiting superior rejection performance.

SEM imaging provides direct and convincing evidence for analyzing membrane fouling resistance and flux behavior, enabling correlation between macroscopic performance and microscopic morphology. As shown in Figure 14, the surface of the pristine NF270 membrane is smooth and uniform, free of visible cracks or impurities, with small pore structures that require high magnification for clear observation. This structural characteristic contributes to its strong size exclusion capability, allowing effective retention of larger organic molecules and resulting in superior rejection performance.

**Figure 15:**
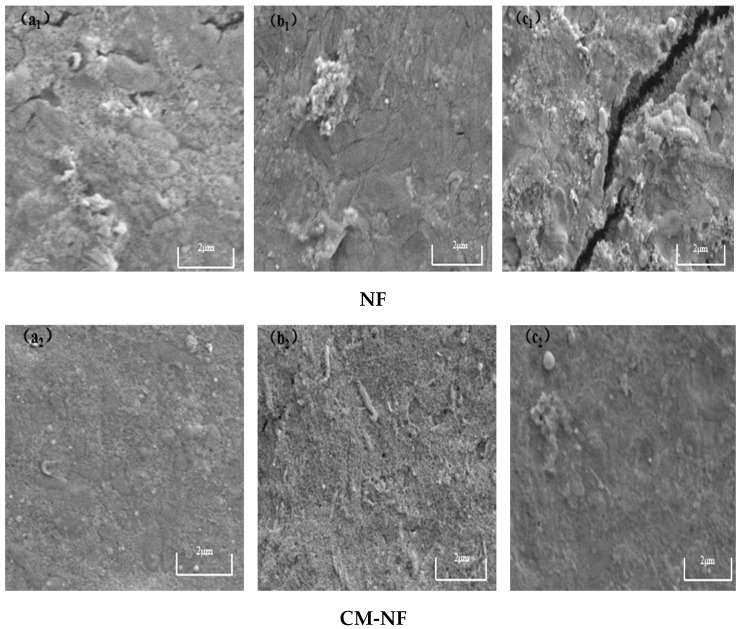
SEM images of the fouling layer on the surface of NF membrane under different HA concentrations. (**a_1_**) 5 mg/L; (**b_1_**) 10 mg/L; (**c_1_**) 20 mg/L—Direct NF vs. (**a_2_**) 5 mg/L; (**b_2_**) 10 mg/L; (**c_2_**) 20 mg/L—CM-NF.

**Figure 16:**
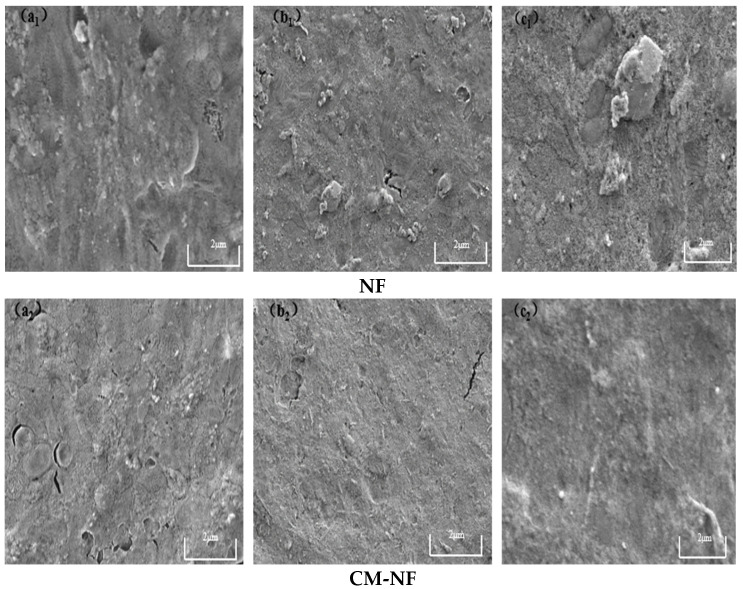
SEM images of the fouling layer on the surface of NF membrane under different SA concentrations. (**a_1_**) 5 mg/L; (**b_1_**) 10 mg/L; (**c_1_**) 20 mg/L—Direct NF vs. (**a_2_**) 5 mg/L; (**b_2_**) 10 mg/L; (**c_2_**) 20 mg/L—CM-NF.

**Figure 17:**
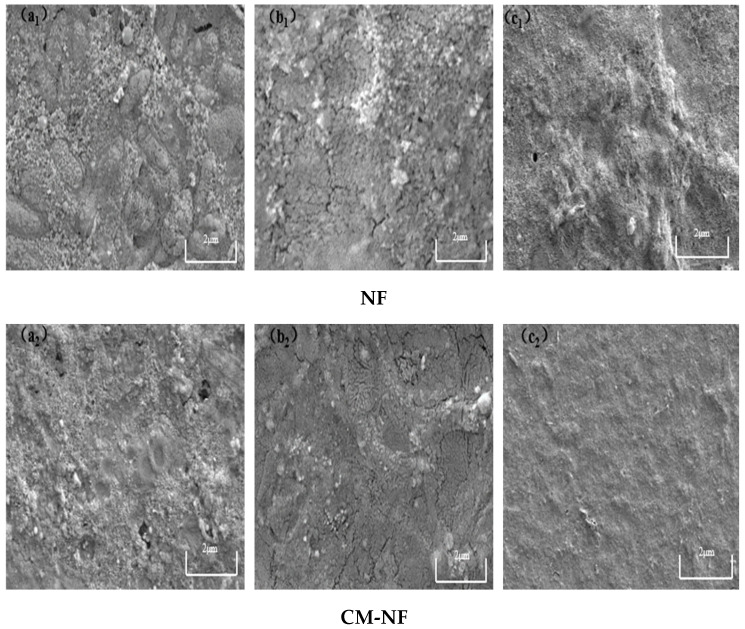
SEM images of the fouling layer on the surface of NF membrane under different BSA concentrations. (**a_1_**) 5 mg/L; (**b_1_**) 10 mg/L; (**c_1_**) 20 mg/L—Direct NF vs. (**a_2_**) 5 mg/L; (**b_2_**) 10 mg/L; (**c_2_**) 20 mg/L—CM-NF.

Although both direct NF and the CM–NF process experience membrane fouling—with severity increasing at higher concentrations of HA, SA, and BSA—fundamental differences are observed in fouling morphology, evolution, and extent. As shown in Figure 15, Figure 16 and Figure 17, in direct NF, pollutants directly form a dense and hardly removable cake layer. This is manifested as bulky HA aggregates, gel-induced scaling from SA–Ca^2+^ complexes, and a dense coverage layer by BSA, leading to severe and largely irreversible fouling.

**Figure 18:**
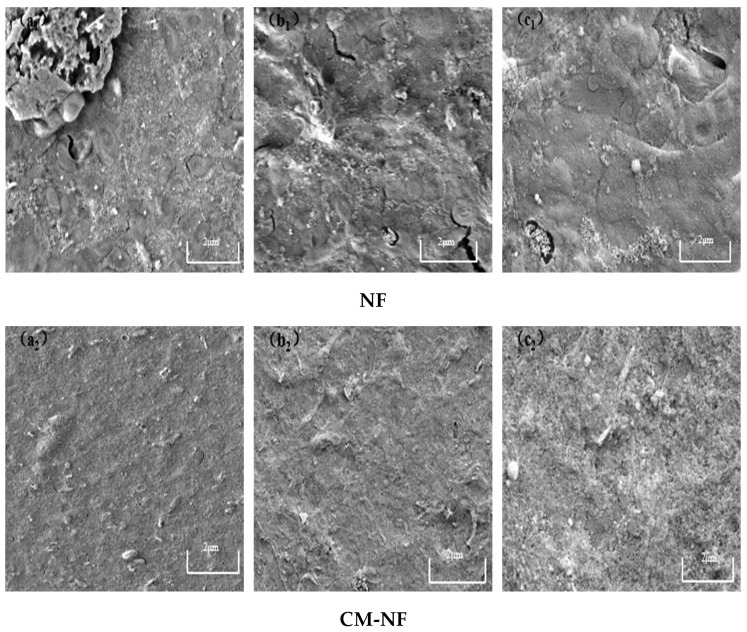
SEM images of the fouling layer on the surface of NF membrane under different Ca^2+^ concentrations. (**a_1_**) 80 mg/L; (**b_1_**) 120 mg/L; (**c_1_**) 160 mg/L—Direct NF vs. (**a_2_**) 80 mg/L; (**b_2_**) 120 mg/L; (**c_2_**) 160 mg/L—CM-NF.

In contrast, elevated Ca^2+^ levels were observed to alleviate membrane fouling. As illustrated in Figure 18, under direct NF at low Ca^2+^ concentration, the membrane was covered by densely distributed granular contaminants and large aggregates, whereas at high Ca^2+^ levels, foulants showed a ridge-like distribution with minor cracks and a relatively loose cake layer structure. In comparison, the CM–NF process—benefiting from ceramic membrane pretreatment—significantly optimizes the fouling layer structure, maintaining it in a loose, porous, and relatively uniform state. This structural advantage not only reduces fouling severity but also enables partial foulant removal through hydraulic flushing, demonstrating enhanced antifouling performance and operational stability.

## 4. Conclusions

Across various pollutant concentration ranges, the integrated CM–NF process demonstrated superior performance in removing organic pollutants and inorganic ions from water compared to the standalone NF process. The combined system achieved average removal rates of 95.60% for DOC, 98.55% for UV_254_, 34.50% for conductivity, and 50.71% for Ca^2+^. Notably, it improved the removal efficiency of conductivity and Ca^2+^ by approximately 10% and 16%, respectively, over the direct NF process. Under HA-dominated conditions in particular, the Ca^2+^ removal rate of the CM–NF process reached 82.25%, nearly 50% higher than that of NF alone.

Variations in pollutant concentration significantly influenced the filtration performance of the NF membrane. As the concentrations of HA, BSA, and SA increased, the specific flux of the NF membrane gradually declined, while the total fouling resistance rose accordingly. In contrast, when the Ca^2+^ concentration increased beyond a certain threshold, it helped alleviate membrane fouling to some extent through a bridging effect that modified the fouling layer structure. CM pretreatment markedly enhanced the specific flux of the NF membrane, with the degree of improvement following the order HA > BSA > SA, and simultaneously reduced the proportion of irreversible fouling. SEM image analysis revealed that high concentrations of HA, BSA, and SA led to the formation of a denser cake layer on the NF membrane surface. In the presence of Ca^2+^, bridging effects promoted the aggregation of pollutants into structurally looser complexes, thereby improving cake layer morphology. Compared to the direct NF process, the CM–NF process significantly reduced the coverage density and thickness of the fouling layer, effectively mitigating the extent of membrane fouling.

## Figures and Tables

**Table 1:** Experimental Water Parameter Table.

Water Quality Condition	Test Group	HA/ mg/L	SA/ mg/L	BSA/ mg/L	CaCl_2_/ mg/L
**NOM** **Concentration** **Effect**	a	5	10	10	120
		10	10	10	120
		20	10	10	120
	b	10	5	10	120
		10	10	10	120
		10	20	10	120
**Ca^2+^** **Concentration** **Effect**	c	10	10	5	120
		10	10	10	120
		10	10	20	120
	d	10	10	10	80
		10	10	10	120
		10	10	10	160

## Data Availability

The original contributions presented in this study are included in the article. Further inquiries can be directed to the corresponding author.
